# Hair follicle associated pluripotent (HAP) stem cells jump from transplanted whiskers to pelage follicles and stimulate hair growth

**DOI:** 10.1038/s41598-022-25383-z

**Published:** 2022-12-07

**Authors:** Koya Obara, Jose Reynoso, Yuko Hamada, Yusuke Aoki, Yutaro Kubota, Noriyuki Masaki, Yasuyuki Amoh, Robert M. Hoffman

**Affiliations:** 1 AntiCancer, Inc., 7917 Ostrow Street, San Diego, CA 92111 USA; 2 Department of Surgery, University of California San Diego, San Diego, CA 92103 USA; 3 Department of Dermatology, Kitasato University School of Medicine, Minami Ward, Sagamihara, Kanagawa 252-0374 Japan

**Keywords:** Cell biology, Stem cells

## Abstract

Stimulation of hair growth in hair loss has been a difficult goal to achieve. Hair follicle-associated pluripotent (HAP) stem cells express nestin and have been shown to differentiate to multiple cell types including keratinocytes, neurons, beating cardiac muscles and numerous other cell types. HAP stem cells originate in the bulge area of the hair follicle and have been shown to migrate within and outside the hair follicle. In the present study, the upper part of vibrissa follicles from nestin-driven green-fluorescent protein (GFP) transgenic mice, containing GFP-expressing HAP stem cells, were transplanted in the dorsal area of athymic nude mice. Fluorescence microscopy and immunostaining showed the transplanted HAP stem cells jumped and targeted the bulge and hair bulb and other areas of the resident nude mouse pelage follicles where they differentiated to keratinocytes. These results indicate that transplanted nestin-GFP expressing HAP stem cells jumped from the upper part of the whisker follicles and targeted nude-mouse hair follicles, which are genetically deficient to grow normal hair shafts, and differentiated to keratinocytes to produce normal mature hair shafts. The resident nude-mouse pelage follicles targeted by jumping whisker HAP stem cells produced long hair shafts from numerous hair follicles for least 2 hair cycles during 36 days, demonstrations that HAP stem cells can stimulate hair growth. The present results for hair loss therapy are discussed.

## Introduction

Hair follicle associated pluripotent (HAP) stem cells expressing nestin were discovered by our laboratory^1^. HAP stem cells were subsequently shown to differentiated to keratinocytes^2^, neurons^2^, nascent blood vessels^3^, beating cardiac muscle^4^ and many other cell types. Oshima et al. showed that hair follicle stem cells trafficked from the bulge area of the whisker follicle to the hair bulb and formed sebaceous glands of the follicle^5^. We subsequently showed that HAP stem cells also could traffic from the bulge area to the dermal papilla and other parts of the hair follicle in vivo^6^, and in 3-D gelform culture in vitro^7^.

The athymic nu/nu nude mouse is unable to produce mature hair shafts due to altered keratinization due to a mutation in the *FOXN1* gene which causes hair differentiation to fail, resulting in broken hair shafts and the mouse to appear nude^8–11^. In the present study we show that nestin-driven green fluorescent protein (ND-GFP)-expressing HAP stem cells in transplanted upper parts of whisker follicles jump to resident nude-mouse pelage hair follicles and stimulate normal hair growth. The implication of the present results for cellular therapy for hair loss is discussed.

## Results

### HAP stem cells in the upper part of whisker follicles from ND-GFP mice jump from their niche after transplantation to nude mice

**Figure 1:**
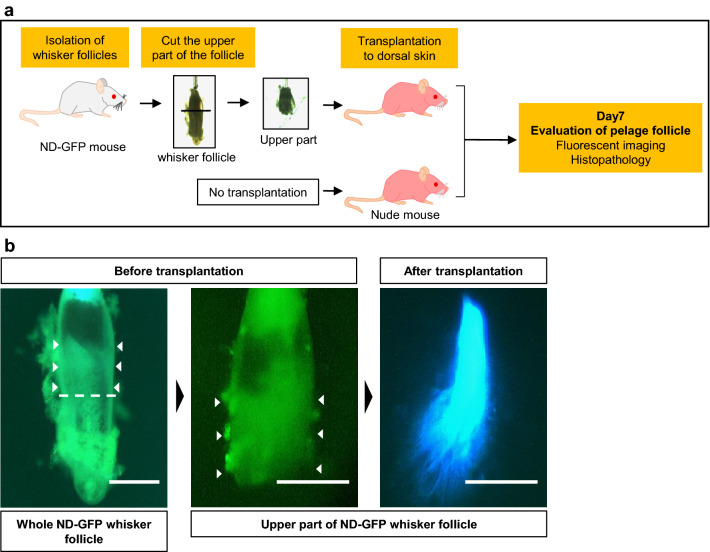
The upper part of whisker follicle from ND-GFP mouse before and after transplantation. (**a**) Schematic representation of experiment. (**b**) Whole-whisker follicle in ND-GFP mice and its upper part before and after transplantation to nude mice. White dotted line indicates cut part of the whole whisker follicle. White arrowheads indicate HAP stem cells in the bulge area. Scale bar = 500 µm.

To demonstrate the effect of HAP stem cell transplantation on hair growth, the upper part of whisker follicles from ND-GFP mice were transplanted into the dorsal skin of nude mice (Fig. 1a). Isolated whisker follicles from ND-GFP mice before transplantation had a high density of HAP stem cells especially in the bulge area (Fig. 1b). Seven days after transplantation of the upper part of whisker follicles from ND-GFP mice to the dorsal skin of nude mice, the skin of transplantation site was isolated and observed subcutaneously under fluorescence microscopy. The entire upper parts of the whisker follicle had high fluorescence due to HAP-stem-cell expressing GFP (Fig. 1b). HAP stem cells jumped from their niche in a fine network pattern of upper part of the hair follicle (Fig. 1b).

### HAP stem cell from transplanted whisker follicles from ND-GFP mice jumped and targeted the bulge area and hair bulb of pelage follicles in nude mice

**Figure 2:**
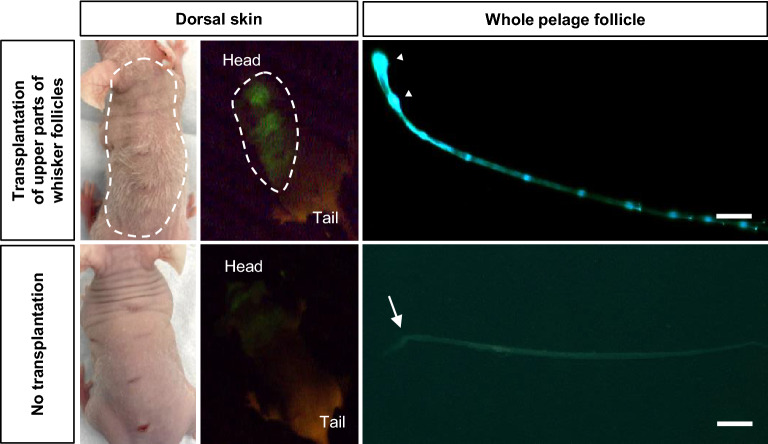
Location of HAP stem cells from the upper part of whisker follicles from ND-GFP mice in nude mice after transplantation. Fluorescence imaging of entire dorsal skin and pelage follicles of nude mice after transplantation of the upper part of whisker follicles from ND-GFP mice. White dotted line indicates hair growth area of the dorsal skin of nude mice. White arrowheads indicate HAP stem cells. White arrows indicate the twisted part of a pelage follicle. Scale bar = 100 µm.

Seven days after transplantation of the upper parts of whisker follicle from ND-GFP mice, hair growth area at the dorsal skin of nude mice was observed by fluorescence imaging. The mice transplanted with upper parts of whisker follicle from ND-GFP mice had numerous long pelage follicles on the entire dorsal skin that expressed GFP from the HAP stem cells consistent with hair growth sites (Fig. 2). Non-transplanted control mice had only a few short pelage follicles on the dorsal skin and did not express GFP. A pelage follicle was removed from the skin of each mouse and observed under fluorescence microscopy. The pelage follicles of the mice transplanted with the upper part of whisker follicle from ND-GFP mice strongly expressed GFP in the bulge area and hair bulb, indicating that jumping HAP stem cells targeted these areas of the pelage follicles (Fig. 2). The HAP stem cells also migrated to the tip of the normal hair shaft produced by the nude-mouse pelage follicles targeted with jumping HAP stem cells (Fig. 2). The pelage follicles of the non-transplanted control mice did not express GFP. The pelage follicles of the mice transplanted with the upper part of whisker follicles from ND-GFP mice were straight, in contrast the pelage follicles of the non-transplanted control mice which were twisted (Fig. 2).

### Pelage follicles of nude mice targeted with HAP stem cells jumping from transplanted whisker follicles of ND-GFP mice had an enlarged isthmus and hair bulb and straightened infundibulum

**Figure 3:**
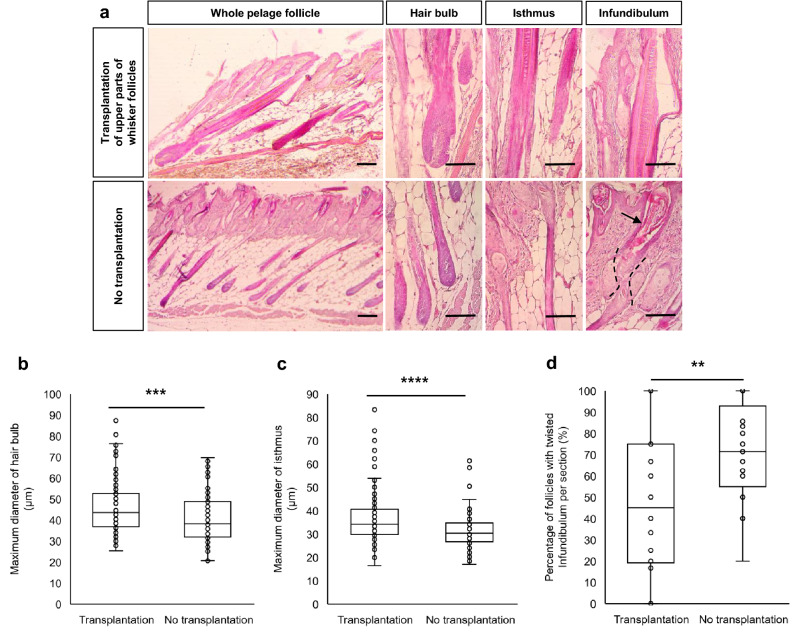
Morphological change of pelage follicle of nude mice after transplantation of upper parts of whisker follicle from ND-GFP mice. (**a**) Hair bulb, isthmus and infundibulum of pelage follicles in nude mice after transplantation of upper part of whisker follicles from ND-GFP mice. Black dotted lines indicate the twisted part of the infundibulum. Black arrows indicates amorphous eosinophilic material within the infundibulum. Scale bar = 50 µm. (**b**) The diameter of hair bulb of nude-mouse pelage follicle 7 days after transplantation of the upper part of whisker follicles from ND-GFP mice, or without transplantation (mean ± SEM = 46.30 ± 1.24 (transplantation), 40.71 ± 0.91 (no transplantation); n = 109 hair follicles, 5 mice transplanted with the upper part of whisker follicles from ND-GFP mice; n = 149 hair follicles, 5 non-transplanted control mice). (**c**) The diameter of isthmus of nude-mouse pelage follicle 7 days after transplantation of the upper part of whisker follicles from ND-GFP mice or without transplantation (mean ± SEM = 36.62 ± 0.87 (transplantation), 31.22 ± 0.77 (no transplantation); n = 140 hair follicles, 5 mice transplanted with upper parts of whisker follicles from ND-GFP mice; n = 101 hair follicles, 3 non-transplanted control mice). (**d**) The percentage of twisted infundibula 7 days after transplantation of upper parts of whisker follicles from ND-GFP mice or without transplantation in nude mice (mean ± SEM = 46.95 ± 6.23 (transplantation), 71.77 ± 4.07 (no transplantation); 153 hair follicles, 5 mice transplanted with the upper part of whisker follicles from ND-GFP mice; 146 hair follicles, 3 non-transplanted control mice). Data are presented as box and whisker plots in (**b**), (**c**), (**d**). Population medians, first and third quartiles are represented by the boxes, maxima and minima by the whiskers, and individual numerical value by the overlaid single points, respectively. Two-sided unpaired Student’s *t-*test was applied for all statistical analyses in this figure. Significance was indicated as **P < 0.01, ***P < 0.001, ****P < 0.0001. Source data are provided as a Source Data file.

Histopathological analysis of the skin from nude mice transplanted with upper parts of ND-GFP mice-whisker follicles and non-transplanted control mice was performed at 7 days after transplantation. In the skin specimen stained with hematoxylin and eosin (H&E), the whole pelage follicles of non-transplanted control mice appeared to be shrunken, although the hair cortex and medulla formation appeared to be normal (Fig. 3a). At the level of the sebaceous gland, the hair shafts and infundibulum were twisted and distorted, and hair shafts contained amorphous eosinophilic material within the infundibulum and broke off at the surface (Fig. 3a). In contrast, the whole pelage follicles of nude mice transplanted with the upper parts of ND-GFP whisker follicles appeared normal compared to the shrunken pelage follicles of the non-transplanted mice, and the hair cortex and medulla also appeared to be normal (Fig. 3a). At the level from the sebaceous gland to the skin surface, many hair shafts and infundibula grew straight compared to the twisted pelage follicles of non-transplanted mice. There was less amorphous eosinophilic material within the infundibulum of the transplanted mice (Fig. 3a). The diameter of hair bulb was significantly larger in the transplanted mice than non-transplanted control mice (*P* = 2.49 × 10^−4^) (Fig. 3b). The diameter of isthmus was significantly larger in the transplanted mice than non-transplanted control mice (*P* = 1.39 × 10^−5^) (Fig. 3c). Pelage follicles with a twisted infundibulum were significantly fewer in the mice transplanted with upper parts of ND-GFP whisker follicles than non-transplanted control mice (*P* = 0.0016) (Fig. 3d).

### Transplanted whisker HAP stem cells jumping to the bulge and hair bulb of nude-mouse pelage follicle differentiated into keratinocytes

**Figure 4:**
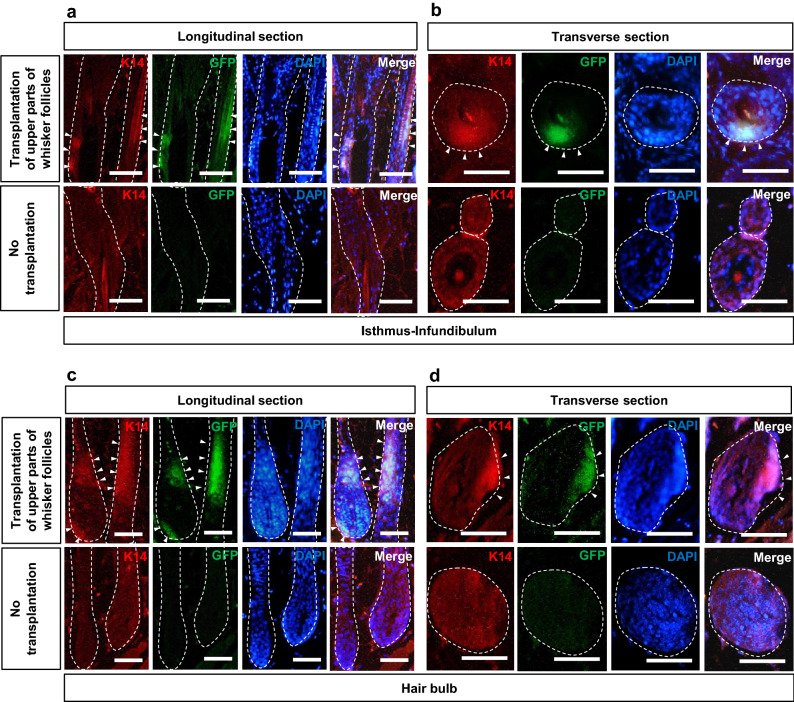
Characteristics of the isthmus and infundibulum, and hair bulb of nude-mouse pelage follicles after transplantation of the upper part of whisker follicles from ND-GFP mice. (**a**) Longitudinal section of a nude-mice pelage follicle from isthmus to infundibulum in the mice transplanted with upper part of whisker follicles from ND-GFP mice or non-transplanted control mice. Scale bar = 50 µm. (**b**) Transverse section of a nude-mice pelage follicle at the isthmus in the transplanted mice or non-transplanted control mice. Scale bar = 25 µm. (**c**) Longitudinal section of a nude-mice pelage follicle at the hair bulb in the transplanted mice or non-transplanted control mice. Scale bar = 50 µm. (**d**) Transverse section of nude-mice pelage follicle at the hair bulb in the transplanted mice or non-transplanted control mice. Scale bar = 25 µm. White dotted lines outline the pelage follicle. White arrowheads indicate the HAP stem cells which jumped to the pelage follicle.

**Figure 5:**
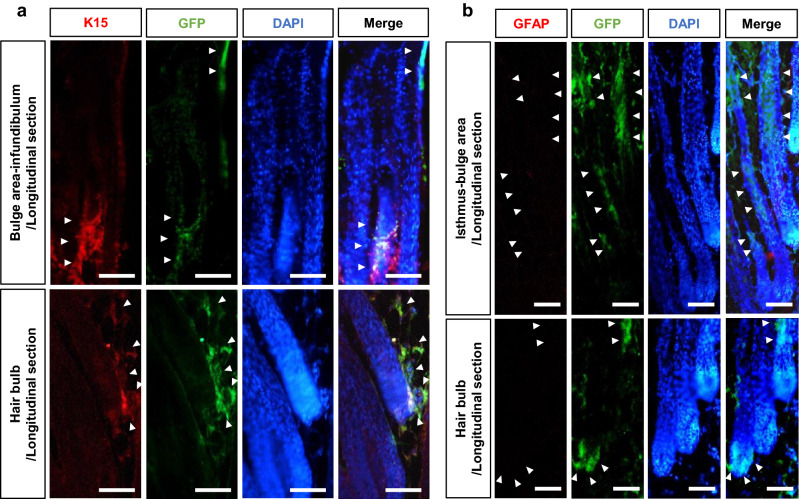
Characteristics of the isthmus, infundibulum and hair bulb stained with GFP and K15 or GFAP of nude-mouse pelage follicle after transplantation of the upper part of whisker follicles from ND-GFP mice. (**a**) Longitudinal section stained with K15 and GFAP of the bulge area and infundibulum (upper row), and hair bulb (lower row) of nude-mouse pelage follicle in the mice transplanted with upper part of whisker follicles from ND-GFP mice. (**b**) Longitudinal section stained with GFP and GFAP of the isthmus and bulge area (upper row), and hair bulb (lower row) of nude-mouse pelage follicle in the transplanted mice. Scale bar = 50 µm. White arrowheads indicate the HAP stem cells which jumped to the pelage follicle.

To confirm location and property HAP stem cells which targeted the pelage follicle in the nude mice, immunofluorescence staining was performed histologically. The isthmus and infundibulum of pelage follicles of mice transplanted with upper parts of ND-GFP whisker follicles expressed GFP from the outer root sheath to the inner root sheath (Fig. 4a,b). Cytokeratin 14 (K14) was strongly expressed at GFP-positive sites in the isthmus and infundibulum of pelage follicles of the transplanted mice (Fig. 4a,b). The isthmus and infundibulum of pelage follicles of non-transplanted control mice did not strongly express K14. The hair bulb of pelage follicles of transplanted mice expressed GFP from the outer root sheath to the inner root sheath (Fig. 4c,d). K14 was strongly expressed at GFP-positive HAP stem cells in the hair bulb of pelage follicles of the transplanted mice (Fig. 4c,d). The hair bulb of pelage follicles of non-transplanted control mice did not strongly express K14. GFP-positive sites in the bulge area, infundibulum and hair bulb of pelage follicles weakly positive for K15 and negative for glial fibrillary acidic protein in the transplanted mice (GFAP) (Fig. 5a,b), unlike cultured HAP stem cells where were positive for both K15 and GFAP^2^.

### Transplanted upper parts of whisker follicles from C57BL/6 J mice promoted nude-mouse pelage hair growth

**Figure 6:**
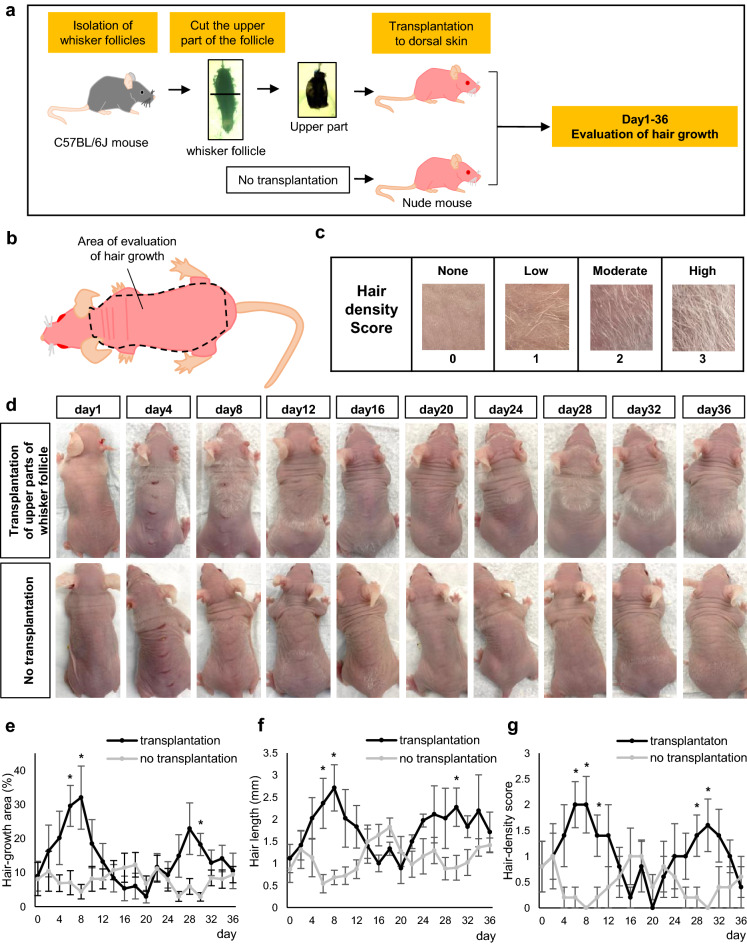
Hair growth of nude-mouse pelage follicles stimulated by transplanted upper part of whisker follicles from C57BL/6 J mice. (**a**) Schematic representation of experiment. (**b**) Schematic representation of the range of hair growth evaluation in nude mice. (**c**) Hair density score in nude mice. (**d**) Time course of hair growth of the nude mice transplanted with the upper part of whisker follicles from C57BL/6 J mice or nude mice without transplantation. (**e**) Time course of hair-growth area of transplanted mice or non-transplanted control mice (n = 5 for each group). (**f**) Time course of hair length of mice transplanted with the upper part of whisker follicles from C57BL/6 J mice or non-transplanted control mice (n = 5 for each group). (**g**) Time course of hair density score of transplanted mice or non-transplanted control mice (n = 5 for each group). Two-way ANOVA followed by Bonferroni’s multiple comparisons post-hoc test was applied for all statistical analyses in this figure. Values are expressed as the mean ± SEM. Significance was indicated as *P < 0.05. Source data are provided as a Source Data file.

To analyze the efficacy of hair growth with HAP stem cell transplantation, the upper parts of the whisker follicles from C57BL/6 J mice were transplanted to the dorsal skin of nude mice (Fig. 6a). The evaluation of hair growth in the nude mice comprised for the hair-growth area, hair length and hair-density score (Fig. 6b,c). The transplanted nude mice had extensive pelage hair growth on the dorsal skin by day 6 after transplantation. The pelage hair shafts had extended longer and spread on the entire dorsal skin by day 8 after transplantation. (Fig. 6d). In contrast, the nude mice without transplantation did not grow normal hair shafts (Fig. 6d). The hair-growth area significantly increased over time in the transplanted mice compared to non-transplanted control mice (*P* = 0.0060 at day 6; *P* = 0.0099 at day 8) (Fig. 6e). The hair length significantly increased over time in the transplanted mice compared to non-transplanted control mice (*P* = 0.0084 at day 6; *P* = 0.0030 at day 8) (Fig. 6f). The hair-density score significantly increased over time in the transplanted mice compared to non-transplanted control mice (*P* = 0.0031 at day 6; *P* = 0.0032 at day 8; *P* = 0.0139 at day10) (Fig. 6g). From day 10 to day 20 in the transplanted mice, the hair-growth area, hair length and hair-density score decreased and then hair growth resumed peaking at day 30, compared to non-transplanted control mice. The hair-growth area (*P* = 0.0011 at day 30), hair length (*P* = 0.0147 at day 30) and hair-density score (*P* = 0.0139 at day 28; *P* = 0.0069 at day 30) were increased in the transplanted mice compared to non-transplanted control mice (Fig. 6d–f). Thus, the mice transplanted with the upper parts of whisker follicles went through at least hair two cycles (Fig. 6d–f).

## Discussion

Promoting hair growth through cell therapy has been attempted for a long time, but with limited success^12^. Pioneering studies demonstrated that it was possible to promote hair follicle neogenesis with the transplantation of rat vibrissa dermal papillas^13,14^. Since then, hair follicle induction by isolated mouse dermal papillae and dermal sheath cells was shown to be enhanced in combination with epithelial cells^15^. McElwee et al. reported that cells derived from the peribulbar dermal-sheath cup induced new hair follicles and that transplantation of these cells into mice induced hair growth, associated with dermal papilla formation^16^. Rahmani et al. reported that self-renewing hair-follicle dermal-stem cells, located in the dermal sheath, regenerated the dermal sheath, populated the dermal papilla with new cells and modulated hair growth and hair type^17^. These discoveries showed the possibility of cellular therapy for hair follicle regeneration. Moreover, it has been reported that spheroids formed from dermal papilla cells from rodents or humans were able to induce hair follicle neogenesis^18–20^. Spheroids formed from dermal papilla cells also demonstrated their capacity to support the formation of hair follicles, especially in combination with newborn mouse epidermal cells^21^ or interfollicular keratinocytes^22^. Recently, a clinical trial of injected dermal-sheath-cup cells in male and female patients with pattern alopecia has been also carried out with moderate and temporary hair growth stimulation^23^.

In the present study we showed transplantation of the upper part of the whisker follicles, each containing numerous HAP stem cells, stimulated nude-mouse pelage hair growth. This is first report that the upper part of hair follicles containing the bulge area stimulated pelage hair growth upon transplantation. HAP stem cells from the transplanted upper parts of ND-GFP whisker follicles jumped to nude-mouse pelage follicles in the hair bulb and bulge and stimulated hair growth with normal hair follicle morphology. HAP stem cells that jumped to nude-mouse pelage follicles expressed K14 and weakly expressed K15 and did not express GFAP. We previously demonstrated previously that HAP stem cells differentiated in culture to GFAP-positive glial cells and K15-positive keratinocytes^2^. The present result suggests that HAP stem cells that jumped to nude-mouse pelage follicles were differentiated to express K14 and K15 but no longer expressed GFAP (Figs. 4, 5).

Nude mice have about the same number of hair follicles as wild-type mice^10^, and the follicle grow actively in cycles^8,24,25^. However hair fibers in nude mice break easily because the cuticle is not normal^8–11^. Thus, alterations in keratinization appear to be the primary mechanism for the development of the nude mice phenotype. The present study showed that HAP stem cells from transplanted with the upper part of whisker follicles could jump to nude-mouse pelage follicles and differentiate to keratinocytes, to produce normal-appearing hair. At the same time, the jumping HAP stem cells induced an enlargement of the nude-mouse pelage follicles, which thereby acquired a normal morphology.

Zhang et al. showed that epithelial cells leak out from the hair follicle stem cell area into the dermis which contributed to the miniaturization of hair follicles in aging mice^26^. The present result suggests the possibility of treating aging hair loss with HAP-stem-cell transplantation.

Other studies have shown that hair follicle germs are formed from stem cells which were transplanted to treat hair loss. Asakawa et al. reported that ectopic transplantation of a bioengineered hair-follicle germ, constructed with epithelial and mesenchymal cells derived from embryonic skin, resulted in the development of normal hair follicles^27^. However, there are ethical issues involved in the clinical application of fetal cells. Takagi et al. created a bioengineered three-dimensional integumentary organ system with accessory organs such as sebaceous glands and hair follicles, from induced pluripotent stem cells (iPSCs)^28^. The problem with iPSCs is that they can form teratomas^29–31^. HAP stem cells used in the present study are readily accessible from everyone, and can be used autologously without immune-suppression, do not form tumors, and can be cryopreserved without loss of pluripotency, allowing individualized banking^2,32,33^. The reason why the upper part of whisker follicles was transplanted was because we expected a continuous supply of HAP stem cells to be produced. In the future, the effect on hair growth after subcutaneous injection of HAP-stem-cell suspensions will be studied.

**Figure 7:**
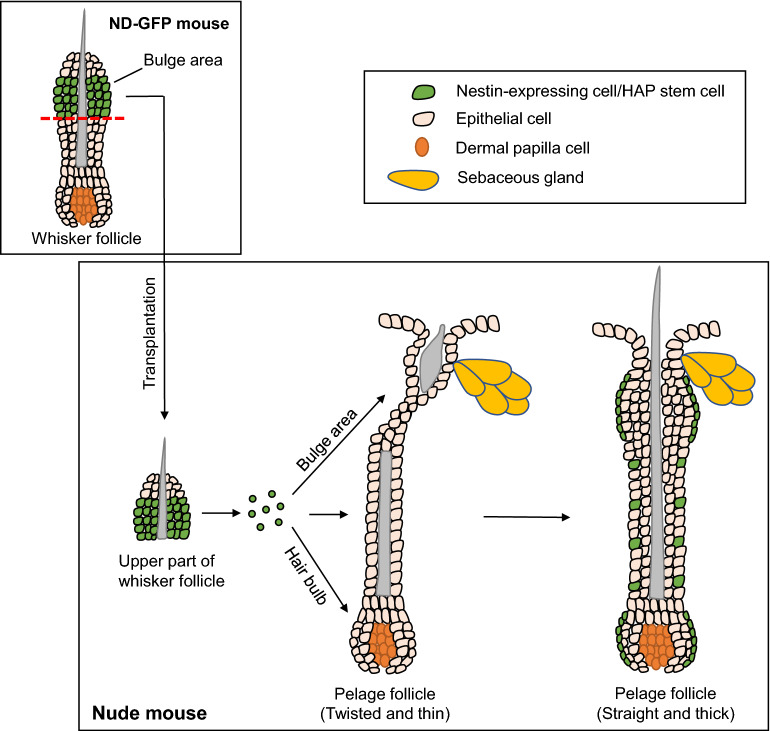
Illustration of hair-growth stimulation of nude mice after transplantation of HAP stem cells. When the upper part of whisker follicles of ND-GFP mice was transplanted to nude mice, HAP stem cells expressing GFP jumped and targeted nude-mouse pelage follicles, mainly in the bulge and hair bulb where they differentiated to keratinocytes. As a result, pelage follicles of nude mice become wider and the twisted infundibulum was straightened, and extensive and long-term hair growth of nude mice was stimulated.

The present study suggests that HAP stem cells in the upper part of transplanted whisker follicles from C57BL/6 J mice jumped from the bulge area of the whisker follicles to target the abnormal pelage follicles of nude mice which then produce normal long hair shafts, while acquiring a normal hair-follicle morphology. The nestin-GFP label of the HAP stem cells enabled the observation of the jumping of HAP stem cell from the upper part of normal whiskers, to target the bulge area and hair bulb of resident nude-mouse pelage follicles, differentiating to keratinocyte to produce normal-like hair shafts (Fig. 7). Future studies will involve testing transplanted HAP stem cells for their hair-growth stimulating efficacy in mouse models of androgenic alopecia (AGA) and aging-hair loss, as the present study has demonstrated that HAP stem cells can stimulate hair growth. Militzer showed that the regional distribution of the anagen hair pattern in nude mice are due to a wave-like pattern of hair generation^25^, which also appears to occur in the nude mice with hair-growth promotion due to transplantation of the upper part of whisker follicles. In addition, the transplanted HAP stem cells that have jumped to the nude-mouse pelage follicles may be cyclically active in promoting hair growth, with periods of inactivity, or even loss of hair-promoting-capability. Therefore, repeated transplantation of HAP stem cells may be necessary to maintain hair-growth promotion, which may be a clinical limitation in the future.

## Methods

### Animals

All mice were used under an AntiCancer Inc. Institutional Animal Care and Use Committee (IACUC) protocol that was specifically approved for the present study, which followed the principles and procedure outline in the National Institutes of Health Guide for the Care and Use of Animals under Assurance Number A3873-1. All mouse experiments were in accordance with animal welfare laws, complied with ARRIVE guidelines. Athymic “nude” mice at 4 weeks of age and C57BL/6 J mice at 4 weeks of age, as well as ND-GFP mice at 4 weeks of age from AntiCancer Inc. (San Diego, CA, USA) were used in the present study. In order to minimize any suffering of the animals, they were anesthetized by subcutaneous injection of anesthesia and analgesics, which consist of 20 mg/kg ketamine, 15.2 mg/kg xylazine, and 0.48 mg/kg acepromazine maleate, for all surgical experiments.

### Isolation of whisker follicles from the whisker pad of mice

All surgical procedures were carried out in a sterile environment. ND-GFP mice or C57BL/6 J mice, 4 weeks old were anesthetized with ketamine prior to the procedure. The upper lip, containing the whisker pad was cut and its inner surface exposed. Intact whisker follicles were dissected under a binocular microscope^34^. The whisker follicles from the pad were plucked by pulling them gently with a fine forceps and fine needle. Whisker follicles were divided into three equal parts with a fine needle under a binocular microscope and upper parts were isolated^34^. All follicles were then kept in PBS.

### Transplantation of the upper part of whisker follicles from ND-GFP or C57BL/6 J mice to nude mice

Isolated upper parts of whisker follicles of ND-GFP transgenic mice or C57BL/6 J mice were transplanted into the dorsal subcutaneous area in 4-week-old nude mice. Nude mice were anesthetized with ketamine prior to the procedure. Five 5-mm transverse full-thickness incisions were made with a scissors vertically on the dorsal skin of the nude mice. Two whisker follicle upper fragments were placed subcutaneous area in each incision. Non-transplanted control mice similarly had five 5-mm incisions only.

### Evaluation of hair growth

The entire dorsal skin of nude mouse transplanted with the upper part of whisker follicles from C57BL/6 J mice or without transplantation were photographed with a digital camera once every two days for 36 days after transplantation. The hair-growth area, hair length and hair density were measured after transplantation. Each measurement evaluated the entire dorsal skin of nude mice as shown in Fig. 6b. The areas of the head and extremities were excluded from efficacy evaluation. The hair-growth area was calculated as the area growing hair with hair length of 1 mm or longer and a hair density score of 1 or more as a % of the entire dorsal skin of nude mice. To evaluate hair length, 3 hairs were selected randomly and measured for hair length (mm). The average value was calculated from the length of the 3 hairs. The hair density score was determined based on the images of Fig. 6c. The area for evaluation of the hair-density score required hair length of 1 mm or higher, and a hair growth-area of 10% or more. The hair-density score was measured at the location of highest hair density per mouse. The analysis of hair-growth area and hair length was performed using ImageJ software (version 1.52; National Institutes of Health, MD, USA).

### Fluorescence imaging

The observation of mouse whisker and pelage follicle by fluorescence microscopy was done with an IMT-2 inverted microscope (Olympus, Tokyo, Japan) equipped with a mercury lamp power supply. The microscope had a GFP filter set (Chroma Technology, VT, USA). Whole-body fluorescence imaging was done with a FluorVivo™1.0 (INDEC BioSystems, Santa Clara, CA, USA).

### Histology and immunohistochemistry

A hair growth area of the dorsal skin of nude mice was biopsied with a diameter of 5 mm for histological analysis 7 days after transplantation of upper parts of whisker follicle from ND-GFP mice. The excised nude-mouse skin samples were formalin-fixed and paraffin-embedded-blocks (FFPB) were made. The FFPB Sections (4 µm each) were placed on slides and stained with hematoxylin and eosin (H&E), or immunofluorescence staining. For immunofluorescence staining, FFPB sections on slides were incubated with anti-GFP rabbit polyclonal antibody (1:1000, cat #NB600, Novus Biologicals, CO, USA), anti-cytokeratin 14 (K14) mouse monoclonal antibody (1:20, cat #VP-C410, Vector Laboratories, CA, USA), anti-cytokeratin 15 (K15) mouse monoclonal antibody (1:1600, cat #MS-1068, Thermo Scientific, CA, USA) and anti-glial fibrillary acidic protein (GFAP) mouse monoclonal antibody (1:500, cat #MS-280, Lab Vision, CA, USA). These sections were then incubated with goat anti-rabbit IgG conjugated with Alexa Fluor 488® (1:400, Molecular Probes, cat #A11008, OR, USA), goat anti-mouse IgG conjugated with Alexa Flour 568® (1:400, cat #A11004, Molecular Probes) and 4’,6-diamino-2-phenylindole, dihydrochloride (DAPI) (1:5000, cat #SE196, Dojindo, Kumamoto, Japan). The observation of skin specimens with H&E staining was done with an Olympus BH-2 microscope (Olympus). The observation of skin specimens with immunofluorescence staining was done with an IMT-2 inverted microscope (Olympus) which had a GFP filter set (Chroma Technology).

Quantitative analysis of morphology of hair follicles was performed histologically. The maximum diameter of the hair bulb and isthmus of the pelage follicles was measured in H&E-stained sections at 100 × magnification. The diameter of the hair bulb and isthmus of pelage follicles was measured in two sections per skin specimens from nude mice. To count the number of hair follicle with a twisted infundibulum, longitudinal sections of pelage follicles were observed with H&E-stained sections at 100 × magnification. The percentage of hair follicles with a twisted infundibulum was determined with each field of view in two sections per skin specimens from nude mice. Quantitative analysis of the maximum diameter of the hair bulb and isthmus was performed using ImageJ software (version 1.52; National Institutes of Health).

### Statistical analysis

All experimental data are expressed as the mean ± SEM. The differences between groups with all histological analysis were analyzed with the two-sided unpaired Student’s *t* test. Two-way ANOVA followed by Bonferroni’s multiple comparisons post-hoc test was used was used to examine the differences between groups in assessments of hair growing are, hair length and hair density score. Data were analyzed by using Excel with Microsoft 365. A probability value of *P* ≤ 0.05, *P* ≤ 0.01, *P* ≤ 0.001 and *P* ≤ 0.0001 is considered significant.

## Supplementary Information

Supplementary Information.

## Data Availability

Source data for all graphs in main text are provided in the Source Data file. Source data are provided with this paper. Other data are available from corresponding author upon request.
